# Immunoassays for the detection and differentiation of *Paenibacillus larvae*, the etiological agent of American foulbrood (AFB) in honey bees

**DOI:** 10.1038/s41598-026-35590-7

**Published:** 2026-01-20

**Authors:** Antonia Reinecke, Josefine Göbel, Elke Genersch

**Affiliations:** 1 Department of Molecular Microbiology and Bee Diseases, Institute for Bee Research, Friedrich-Engels-Str. 32, 16540 Hohen Neuendorf, Germany; 2 Department of Veterinary Medicine, Institute of Microbiology and Epizootics, Freie Universität Berlin, 14163 Berlin, Germany

**Keywords:** American foulbrood, *Paenibacillus larvae*, Diagnostic, Point-of-care test, Immunoassay, LFA, ELISA, Biological techniques, Biotechnology, Microbiology

## Abstract

The Western honey bee (*Apis mellifera*) is among the most important commercial pollinators in agriculture, but also plays a central role as pollinator in natural ecosystems. The globally occurring brood disease American foulbrood (AFB), caused by the gram-positive, spore-forming bacterium *Paenibacillus larvae*, poses a serious threat to colony health and productivity. Early and accurate diagnosis is therefore essential to effectively contain disease outbreaks. This study aimed to develop a rapid, sensitive point-of-care test in lateral flow format for the simultaneous detection and differentiation of the clinically relevant *P. larvae* genotypes ERIC I and II. To achieve this aim, two target antigens were selected, produced recombinantly or purified, and used to generate monoclonal antibodies. These antibodies formed the basis for the development of two sandwich ELISAs and, building on this, a duplex lateral flow assay. Both immunoassay formats were evaluated with infected larvae samples. The results showed high specificity, sensitivity, and accuracy, allowing reliable detection of *P. larvae* and differentiation between the genotypes ERIC I and II, thereby providing a valuable addition to existing diagnostic methods. In particular, the lateral flow assay enables rapid on-site diagnosis, facilitating timely intervention, and thus supports effective measures to control the spread of the disease.

**Supplementary Information:**

The online version contains supplementary material available at 10.1038/s41598-026-35590-7.

## Introduction

 Like all other living creatures, honeybees are susceptible to infectious diseases. These diseases caused by viruses, bacteria, fungi, or parasites, impair the vitality of bee colonies^1^. The prerequisite for targeted control or treatment of infectious diseases is, first and foremost, the correct diagnosis of the disease, including identification of the pathogen. The development of diagnostic procedures and methods is therefore crucial for improving bee health and ensuring the ecosystem service provided by honey bee colonies, i.e., the pollination of many flowering plants^2–4^.

One of the most important and devastating infectious diseases affecting the Western honey bee (*Apis mellifera*) is a brood disease called American foulbrood (AFB), which not only kills the brood of a colony but also the entire colony as the disease progresses. AFB is caused by the gram-positive, spore-forming bacterium *Paenibacillus larvae* (*P. larvae*)^5^. Since the pathogen is highly infectious and contagious, the disease spreads very quickly within a colony and between colonies. Therefore, AFB is listed as a notifiable animal disease in many countries.

The species *P. larvae* is divided into different phenotypically distinct ERIC genotypes, which were originally defined using repetitive element PCR (repPCR) and primers that amplify enterobacterial repetitive intergenic consensus (ERIC) elements – hence the ERIC genotype designation^5,6^. Only two of these genotypes, ERIC I and ERIC II, have consistently been identified as the cause of AFB-outbreaks in diseased colonies worldwide in recent decades^7^. The other genotypes described, ERIC III to ERIC V, are not associated with more recent AFB-outbreaks^5,7–9^. In experimental infection assays, these three genotypes proved to be hypervirulent, which could explain why they have apparently not become established pathogens in the bee population^5,8,9^.

The two *P. larvae* genotypes ERIC I and ERIC II, which are currently driving the global AFB-outbreak situation, differ genetically in their suite of expressed virulence factors^10,11^. These differences result in variations in pathogenesis and contribute to different levels of virulence as phenotypic traits^12–15^. Over the last two decades, we have gathered knowledge on the species- and genotype-specific virulence factors of *P. larvae*^15,16^. The key virulence factor of *P. larvae* is the chitin-degrading protein *Pl*CBP49, a member of the auxiliary activity 10 (AA10) family of lytic polysaccharide monooxygenases (LPMOs)^17^. *Pl*CBP49 breaks down the peritrophic matrix protecting the larva’s intestine, thereby enabling the bacteria to subsequently attack the intestinal epithelium. As key virulence factor, it is secreted by all virulent *P. larvae* strains, regardless of their genotype. For attacking and breaching the midgut epithelium, *P. larvae* ERIC I and ERIC II use different strategies. *P. larvae* ERIC I expresses two AB toxins, Plx1 and Plx2, which attack the epithelial cells and thereby trigger the invasion of the bacteria into the hemocoel of the larva^18^. *P. larvae* ERIC II has no functional toxin genes^10^, but instead expresses a surface layer (S-layer) protein, SplA, as a genotype-specific virulence factor^19^ that is absent in *P. larvae* ERIC I strains^20^. SplA mediates the adhesion of *P. larvae* ERIC II bacteria to the midgut epithelial cells, thereby initiating the destruction of the epithelial barrier via a still elusive mechanism^19^. For both genotypes, the breakthrough through the intestinal epithelium and invasion into the larva’s hemocoel marks the death of the larva^21^, which is then decomposed by *P. larvae* into a ropy mass. This ropy mass eventually dries into the so-called foulbrood scale, which contains millions of infectious *P. larvae* spores that, when ingested by young larvae, enable the disease to spread throughout the colony. Ropy masses and foulbrood scales are considered sufficiently specific clinical symptoms of AFB to raise suspicion of an AFB outbreak^22^. However, for a definitive diagnosis of AFB, it is necessary to detect the causative agent, *P. larvae*, in appropriate test material from the bee colony suspected to have AFB [reviewed in^23^]. In order to expand the range of detection methods for *P. larvae* and, above all, to have a detection method that can be used as point-of-care test directly on the colony, we developed two novel diagnostic tests, an enzyme-linked immunosorbent assay (ELISA) and a lateral flow assay (LFA). Both are based on the immunodetection of the virulence factors *Pl*CBP49 and SplA for the general detection of the species *P. larvae* and the specific detection of *P. larvae* ERIC II, respectively. Our data presented here for the validation of these assays show that their specificity, sensitivity, and accuracy will make them valuable tools for the diagnosis of AFB.

## Methods

### Bacterial strains and growth conditions

Sixteen *Paenibacillus larvae* strains representing the two *P. larvae* genotypes ERIC I (*n* = 8) and ERIC II (*n* = 8) and different sequence types within the genotypes according to the multi locus sequence typing (MLST) scheme established for *P. larvae*^7^ (Table 1), already characterized in previous studies^7,11,13,24^, were used in this study. The strain ATCC 9545, was obtained commercially from the American Type Culture Collection (ATCC), all other *P. larvae* strains were isolated from brood comb honey of *P. larvae*-infected or AFB-diseased honey bee colonies. In addition, 22 bacterial species, which are commonly found in brood comb honey and hence, become part of the larval feed once the glandular secretions of the nurse bees are supplemented with honey, were isolated from brood comb honey of *P. larvae*-free colonies, identified by sequencing of the 16S rRNA gene^25^, and used as controls in this study (Table 2). *Melissococcus plutonius* type strain DSM 29964^T^, the causative agent of European Foulbrood of honey bees^26^, was obtained commercially from the German Collection of Microorganisms and Cell Cultures GmbH (DSMZ). All vegetative bacteria except *M. plutonius* were cultured on Columbia Sheep blood Agar (CSA) plates (Oxoid Deutschland GmbH, Wesel, Germany; containing 5% sterile defibrinated sheep blood) at 37 °C. Liquid cultures were prepared using Mueller-Hinton-yeast-phosphate-glucose-pyruvate (MYPGP) broth and incubated overnight or for 48 h at 37 °C with shaking. The preparation of spore suspensions (where appropriate) and the determination of their concentration by dilution series was carried out according to the method previously described^13,27^. *M. plutonius* was cultured exactly as already described^28^. Briefly, vegetative bacteria were cultured anaerobically for 72 h at 30 °C either in broth medium or on agar plates prepared from DSM 1582 *M. plutonius* medium (13.5 g KH_2_PO_4_, 10.0 g yeast extract, 10.0 g glucose, 10.0 g soluble starch, 1.0 g cysteine hydrochloride, add deionized water to 1000.0 ml, adjust pH to 6.6 with NaOH).

**Table 1:** *P. larvae* strains used representing the two genotypes ERIC I and ERIC II and different sequence types within the genotypes according to the multi locus sequence typing (MLST) scheme established for *P. larvae*^7^.

*P*.* larvae* strains	ERIC genotype	MLST sequence type
ATCC 9545	I	17
01-440	I	15
02-113	I	18
02-120	I	18
02-130	I	17
03-125	I	19
03-159	I	2
DSM 25429	I	20
DSM 25430	II	11
00-1163	II	10
02–009	II	10
03-194	II	11
03-200	II	11
03-522	II	10
03-525	II	10
20–0360	II	10

**Table 2:** Selection of bacteria typically found in brood comb honey^25^ or being associated with (*P. alvei*, *E. faecalis*) or causing European foulbrood of honey bees (*M. plutonius*)^29^.

Concomitant bacteria	Gram-staining	Saprophyte	Spore former
*Bacillus cereus group*	pos.	Yes	Yes
*Bacillus pumilus*	pos.	Yes	Yes
*Bacillus megaterium*	pos.	Yes	Yes
*Bacillus licheniformis*	pos.	Yes	Yes
*Bacillus velezensis*	pos.	Yes	Yes
*Lysinibacillus fusiformis*	pos.	Yes	Yes
*Lysinibacillus sphaericus*	pos.	Yes	Yes
*Lysinibacillus pakistanensis*	pos.	Yes	Yes
*Microbacterium oxydans*	pos.	No	No
*Gordonia polyisoprenivorans*	pos.	No	Yes
*Paenibacillus barengoltzii*	pos.	Yes	Yes
*Paenibacillus glucanolyticus*	pos.	Yes	Yes
*Paenibacillus odorifer*	pos.	Yes	Yes
*Paenibacillus polymyxa*	pos.	Yes	Yes
*Bacillus mycoides*	pos.	Yes	Yes
*Brevibacillus borstelensis*	pos.	Yes	Yes
*Lactobacillus kunkeei*	pos.	No	No
*Sporosarcina sp.*	pos.	Yes	Yes
*Leifsonia sp.*	pos.	No	No
*Arthrobacter sp.*	variable	?	Yes
*Paenibacillus alvei*	pos.	Yes	Yes
*Enterococcus faecalis*	pos.	Yes	No
*Melissococcus plutonius*	pos.	No	No

### Exposure bioassay

To obtain *P. larvae* ERIC I and ERIC II infected larvae for testing the ELISA and LFA, exposure bioassays were performed essentially as previously described^13,30^.

Exposure bioassays with honey bee larvae are not subject to any specific guidelines or regulations in Germany. Briefly, honey bee larvae were collected from AFB-free colonies at the age of about 12 h after egg hatching (L1 stage), exposed to *P. larvae* spores of the strains ATCC 9545 (genotype ERIC I), DSM 25430 or 20–0360 (both genotype ERIC II; Table 1) for 24 h, and reared in the lab for another 13 days as already described. Throughout the experiment (14 days), bee larvae were observed daily and all dead bee larvae and pupae were collected and analyzed for the presence of vegetative *P. larvae* to confirm or refute *P. larvae* infection as cause of death. To this end, one inoculation loop of the carcass was streaked-out on CSA-plates and the growth of *P. larvae* colonies was evaluated after incubation at 37 °C overnight. The growth of vegetative *P. larvae* cells overnight was taken as confirmation of the presence of vegetative *P. larvae* and, hence, successful *P. larvae* infection. *P. larvae* colonies were identified phenotypically and confirmed by PCR analysis using *P. larvae* specific primers^5,31^. The remains of the larval carcasses were either tested directly or stored at -70 °C until use. For testing the sandwich ELISA or LFA, AFB-dead larvae that died as engorged larvae were preferably used. However, for the testing of *P. larvae* ERIC II infected larvae, pool samples of AFB-dead larvae from earlier developmental stages were also used.

### Field sample material

Field sample material for validating the LFA was obtained from honey bee colonies of two apiaries with officially (in a state laboratory) diagnosed AFB-outbreaks in Brandenburg, Germany, in 2024. One outbreak was officially determined to be caused by *P. larvae* ERIC I and the other by *P. larvae* ERIC II. A total of 40 dead larvae each were collected from four colonies of the ERIC I-outbreak and from the two colonies affected by the ERIC II-outbreak. Dead larvae not yet or already developed into ropy mass were collected from brood combs and one inoculation loop of each larval carcass was streaked-out on CSA plates. Plates were incubated at 37 °C overnight. *P. larvae* colonies were identified phenotypically and confirmed by PCR analysis using *P. larvae* specific primers^5,31^. To confirm the official genotyping results, additional ERIC-genotyping was performed on each larval carcass collected via repetitive element PCR (repPCR) using ERIC-primers as described^5^. Unobtrusive larvae, that tested negative for *P. larvae* were used as controls. All larval samples were either tested directly or stored at − 70 °C until analysis.

### Purification of native *Pl*CBP49

For the purification of native *Pl*CBP49 (49 kDa), the previously described protocol^17^ for the small-scale affinity purification of *Pl*CBP49 was adapted to a larger volume. Briefly, the *P. larvae* ERIC II strain DSM 25430 was cultured in 2x MYPGP broth for 48 h as described above, followed by centrifugation of the bacterial cultures at 3,200 x *g* at 4 °C for 20 min. The culture supernatant was collected, supplemented with a protease inhibitor (PMSF) (Merck KGaA, Darmstadt, Germany), and subjected to sterile filtration. Supernatants containing *Pl*CBP49 were applied to affinity chromatography on a XK column (Cytiva (formerly GE Healthcare), Massachusetts, USA) packed with chitin beads (New England BioLabs GmbH, Frankfurt am Main, Germany) using a running buffer consisting of 125 mM NaCl, 10 mM CaCl_2_ and 25 mM Tris-HCl (pH 7.5). Using 25 mM acetic acid as eluent, 12 ml fractions were collected at a flow rate of 2.5 ml/min. Increasing the pH value to about 8 with NaOH helped to stabilize the protein. Fractions were checked by SDS-PAGE and those containing *Pl*CBP49 were dialyzed at 4 °C overnight against phosphate buffered saline (PBS). Purity of *Pl*CBP49 was again checked by SDS-PAGE with subsequent Coomassie Staining.

### Recombinant expression and purification of recSplA

For the recombinant production of the S-layer protein SplA^12,19,20^ ((114 kDa) GenBank Accession Number: JQ353714.1), the sequence encoding the SplA protein of *P. larvae* DSM 25430^10^ was PCR-amplified using genomic DNA as template with the primers NdeI_SplA and XhoI_SplA (Supplementary Table S1). The PCR product was cloned into expression vector pET-28a(+) (Novagen, Merck KGaA) using the NdeI and XhoI restriction sites. This cloning strategy resulted in a double methionine codon immediately after the His tag sequence thus preventing the His tag from being translated and added to the expressed protein. The resulting plasmid was designated pET28(+)_*spl*A and successful cloning was confirmed by sequencing the plasmid prior to transforming the plasmid into *E. coli* BL21 cells, resulting in *E. coli*::*spl*A. Expression and purification of the protein from *E. coli*::*spl*A was performed as previously described^19^. In brief, the bacteria pellet was resuspended in 10 ml incubation buffer (50 mM Tris/HCl (pH 7.2), 10 mM MgCl_2_, 0.5% Triton X-100) per gram of wet pellet. Cell lysis was performed with 0.8 g/ml lysozyme per gram wet pellet and 6 M Urea, followed by centrifugation at 5,000 x g at 4 °C for 15 min and subsequent ultracentrifugation of the supernatant at 233,000 x *g* (Beckman Optima MAX-XP benchtop ultracentrifuges and MLA-50 rotor) at 4 °C for 1 h. The recSplA was purified from the concentrated (Amicon cells; Sigma-Aldrich, Merck KGaA) supernatant by gel filtration on a High Load 16/600 Superdex column (Cytiva (formerly GE Healthcare)). Using eluent buffer (50 mM Tris/HCl (pH 7.2), 10 mM MgCl_2_, 6 M urea), 1 ml fractions were collected at a flow rate of 1 ml/min. Fractions were checked with SDS-PAGE and those containing recSplA were pooled and dialyzed at 4 °C overnight against PBS, followed by a precipitation step with 1 M LiCl. The precipitated protein was resolved in PBS and its purity analyzed by SDS-PAGE with subsequent Coomassie Staining.

These antigens were used for the generation of monoclonal antibodies (fzmb GmbH, Bad Langensalza, Germany) directed against *Pl*CBP49 and recSplA^32,33^ as well as for initial testing of the specificity of the generated antibodies in indirect ELISAs by fzmb GmbH. All antibodies were stored at − 20 °C until used.

### Antibody specificity testing via dot blot and western blot analyses

We obtained three monoclonal antibodies each against *Pl*CBP49 (α-*Pl*CBP49 mAb 1, 2, 3) and recSplA (α-SplA mAb 1, 2, 3), which had specifically recognized their respective antigens in an indirect ELISA (fzmb GmbH). The specificity of these antibodies for recognizing the species *P. larvae* (α-*Pl*CBP49 mAbs) or the genotype *P. larvae* ERIC II (α-SplA mAbs) was evaluated in dot blot and western blot analyses. Since SplA is found on the surface of *P. larvae*, the samples used for testing the α-SplA mAbs were bacterial cultures. In contrast, *Pl*CBP49 is a secreted protein and, hence, α-*Pl*CBP49 mAbs were tested using culture supernatants. To obtain culture supernatants, liquid *P. larvae* cultures were centrifuged at 5000 x *g* at 4 °C for 15 min and the culture supernatants were collected.

For dot blot analysis, samples were applied onto a nitrocellulose transfer membrane with a pore size of 0.2 μm (Carl Roth GmbH, Karlsruhe, Germany) using a 96-pocket transfer device (Carl Roth GmbH) providing dots with a 3.2 mm diameter (see supplementary Figure S1A). After blotting, the membranes were cut vertically (one, three or four columns wide, see supplementary Figure S1B, C, D) and full-length membrane pieces were subjected to immunodetection. For western blot analysis, samples were separated by SDS-PAGE using a Mini-PROTEAN TGX Stain-Free Precast Gel (Bio-Rad Laboratories GmbH, Feldkirchen, Germany). The protein bands were then blotted using the Trans-Blot Turbo Mini 0.2 μm Nitrocellulose Transfer Pack and the Trans-Blot Turbo Transfer System (Bio-Rad Laboratories GmbH). Membranes were blocked with TBS buffer (10 mM Tris (pH 7.9), 150 mM NaCl) containing 2% BSA and 5% dry milk for 2 h at room temperature. The membranes were then transferred to TBS-T (TBS with 0.05% Tween 20) containing 5% dry milk and incubated with the primary antibodies (0.01 mg/ml) at 4 °C overnight. An HRP-conjugated goat anti-mouse IgG + IgM antibody (Sigma-Aldrich, Merck KGaA) served as the second antibody (1/10,000 dilution in TBS-T); incubation was performed for 1 h at room temperature. The immunoreactivity was determined using the Clarity Max Western ECL Substrate (Bio-Rad Laboratories GmbH) and evaluated using the ChemiDoc MP Imaging System (Bio-Rad Laboratories GmbH).

### Detection and differentiation of *P. larvae* via sandwich ELISA

Based on the results of the antibody specificity tests described above, two monoclonal antibody pairs directed against *Pl*CBP49 and SplA, respectively, were selected and used to develop and produce two sandwich ELISAs (fzmb GmbH): A *Pl*CBP49-sandwich-ELISA (limit of detection (LOD) < 0.4 ng/ml as determined by titrating purified native *Pl*CBP49 in the sandwich ELISA) based on the selected α-*Pl*CBP49 monoclonal antibodies was established for the detection of the species *P. larvae*, and an SplA-sandwich-ELISA (LOD approx. 4.7 ng/ml as determined by titrating purified recSplA in the sandwich ELISA) based on the selected α-SplA monoclonal antibodies for the specific detection of the genotype ERIC II. Complete ELISA kits together with the information on the LODs were provided by fzmb GmbH for validation. Each ELISA kit included homogenization matrix (ceramic beads), 96-well plates pre-coated with capture antibodies, ELISA-kit sample and ELISA-kit wash buffers, detector antibody conjugate, ELISA Kit substrate solution (TMB), ELISA Kit stop solution (0.5 M H₂SO₄), and positive/negative controls. Validation of the *Pl*CBP49-ELISA and SplA-ELISA was performed with 50 *P. larvae* ERIC I-infected larvae, 50 *P. larvae* ERIC II-infected larvae and 50 non-infected larvae, obtained from exposure bioassays. Infection status of each larva was verified by PCR as described above. Up to two larvae were homogenized in 1000 µl ELISA-kit sample buffer by vortexing with two ceramic beads for 30 s. Larval homogenates and controls were pipetted into the wells of the ELISA-plates (100 µl/well). From each homogenate three aliquots were tested to obtain three technical replicates per sample. The ELISA plates were incubated for 2 h at 37 °C. The homogenates were discarded and the wells were washed using the kit’s ELISA wash buffer (4 × 300 µl/well). The detector antibody conjugate (50 µl/well) was added to the wells and the plates incubated for 1 h at 37 °C, followed by another wash step. ELISA-kit substrate solution (100 µl/well) was added and the plates were incubated for 20 min at room temperature in the dark. The reaction was stopped with 50 µl/well ELISA-kit stop solution. Optical density (OD) was measured at 450/630 nm (Synergy HT, Agilent Technologies (formerly BioTek Instruments), Santa Clara, USA). Values were normalized by subtracting OD_630_ from OD_450_. A normalized OD ≥ 0.1 was considered positive. Tests without valid control results were excluded from analysis. All mean values are given ± standard deviation (SD). The performance of the two sandwich ELISAs was evaluated by comparison with the reference method (PCR). Sensitivity, specificity and accuracy were calculated using a 2 × 2 contingency table. Their respective 95% confidence intervals (95% CI) were determined using the Wilson-Brown method. All statistical analyses were performed within GraphPad Prism 10 (San Diego, CA, USA).

### Detection and differentiation of *P. larvae *via duplex-LFA

The antibody pairs selected for the sandwich-ELISA were also used to develop and produce a duplex-LFA for rapid, point-of-care detection of *P. larvae* and differentiation of the genotypes ERIC I and ERIC II (fzmb GmbH). Complete LFA kits together with the information on the LODs for *Pl*CBP49 (LOD ≤ 15.63 ng/ml) and SplA (LOD < 31.25 ng/ml) were provided by the manufacturer fzmb GmbH for validation. Each kit included homogenization matrix (ceramic beads), a test bottle with LFA-kit sample buffer and a test cassette containing a test stripe (nitrocellulose membrane) with two test lines (T1: immobilized α-*Pl*CBP49 capture antibody; T2: immobilized α-SplA capture antibody) and a control line (immobilized anti-mouse antibody), mounted on a backing card and combined with sample, conjugate, and absorbent pads. The conjugate pad contained the respective α-*Pl*CBP49 and α-SplA detector antibodies conjugated to gold nanoparticles. Validation of the duplex-LFA was performed using 96 *P. larvae* ERIC I-infected larvae and 101 ERIC II-infected larvae from exposure bioassays, as well as 40 ERIC I-infected and 40 ERIC II-infected larvae from field samples collected from AFB-diseased colonies. Negative controls consisted of 101 non-infected larvae from exposure bioassays and 40 non-infected larvae from field samples. Infection status of each larva was verified by PCR as described above. Positive controls were prepared by diluting the respective antigens in LFA-kit sample buffer to 500 ng/µl. The homogenisation of bee larvae was carried out by shaking up to two experimentally infected larvae in a 15 ml bottle containing 5 ml LFA-kit sample buffer and two ceramic beads. For field samples, one larva was used and the larval suspension obtained was diluted 1:2 in LFA-kit sample buffer prior to testing. For each test, 100 µl of the undiluted (experimental infection) or diluted (field sample) homogenate and positive control was applied to the LFA and incubated for 5 min at room temperature. Thereafter, 50 µl (bioassay sample) or 100 µl (field sample) of LFA-kit sample buffer was applied to the LFA and incubation was continued for another 10 min. The signal strength at the control and test lines was colorimetrically quantified using the Cube-Reader (opTricon GmbH (formerly Biosynex Technologies) Berlin, Germany)^34^ and displayed in optical density indices (ODIs). The ODI of 5 was defined as the cut-off point. Tests without a visible or measurable control line were considered invalid. All medians are given with 95% confidence intervals (95% CI). The performance of the duplex-LFA was evaluated by comparison with the reference method (PCR). Sensitivity, specificity and accuracy were calculated using 2 × 2 contingency tables. Their respective 95% confidence intervals (95% CI) were determined using the Wilson-Brown method. All statistical analyses were performed within GraphPad Prism 10 (San Diego, CA, USA).

## Results

### Evaluation of antibody specificity for vegetative *P. larvae*

Three monoclonal antibodies (mAbs), each directed against *Pl*CBP49 (α-*Pl*CBP49 mAb 1, 2, 3) and SplA (α-SplA mAb 1, 2, 3), were commercially produced (fzmb GmbH) using native *Pl*CBP49 and recombinantly expressed recSplA, respectively, as antigens. We tested the specificity of these antibodies for vegetative *P. larvae* using dot blot analyses with eight strains each of *P. larvae* ERIC I and ERIC II (Table 1). Since *Pl*CBP49 is a key virulence factor for the species *P. larvae* and is secreted by all virulent *P. larvae* strains^7,17^, α-*Pl*CBP49 mAbs should be able to react with all *P. larvae* strains. In contrast, SplA is a virulence factor that is specifically expressed by *P. larvae* ERIC II and is absent in *P. larvae* ERIC I strains^19,20^, therefore, α-SplA mAbs should specifically react only with *P. larvae* ERIC II strains. To rule out the possibility that α-*Pl*CBP49 mAbs and α-SplA mAbs cross-react with non-*P. larvae* bacteria present in sample matrices, 20 bacterial species commonly found in brood comb honey (Table 2) were also tested.

**Fig. 1:**
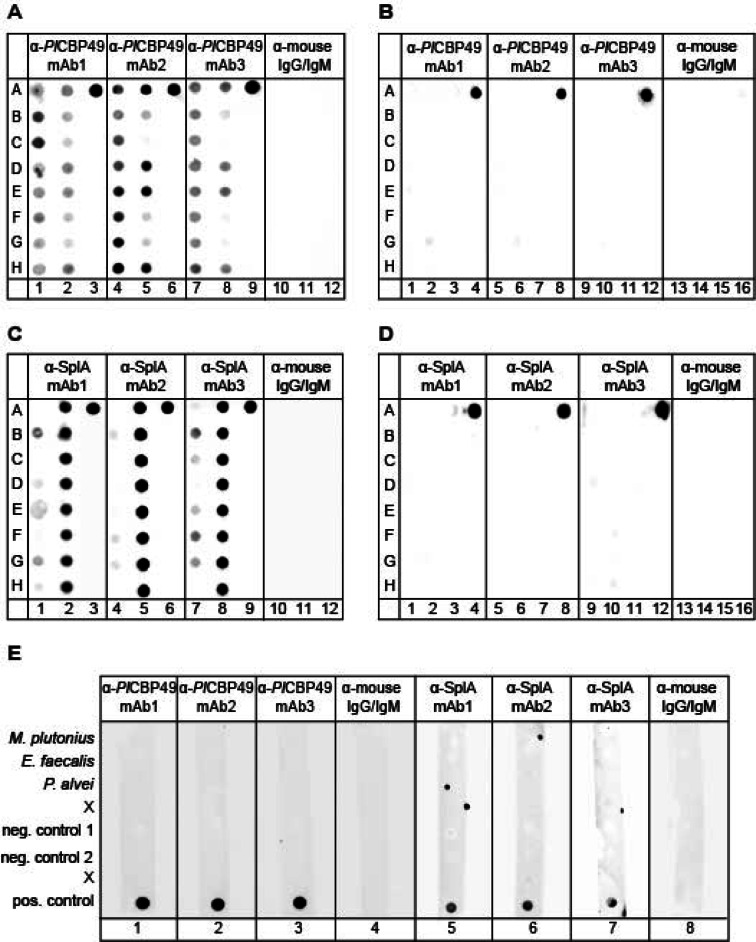
Evaluation of antibody specificity for *P. larvae* by dot blot analysis. (**A**) α-*Pl*CBP49 mAbs tested with eight different *P. larvae* ERIC I (columns 1, 4, 7) and eight different ERIC II strains (columns 2, 5, 8). Native *Pl*CBP49 served as positive control (row A in columns 3, 6, 9) and culture medium as negative control (row B in columns 3, 6, 9). (**B**) α-*Pl*CBP49 mAbs tested with field isolates of 20 different bacterial species commonly isolated from brood comb honey (columns 1, 2, 3, 5, 6, 7, 9, 10, 11). Again, native *Pl*CBP49 served as positive control and culture medium as negative control. (**C**) α-recSplA mAbs tested with eight different *P. larvae* ERIC I strains (columns 1, 4, 7) and ERIC II strains (columns 2, 5, 8). Recombinantly expressed SplA served as positive control (row A in columns 3, 6, 9) and culture medium as negative control (row B in columns 3, 6, 9). (**D**) α-recSplA mAbs tested with field isolates of 20 different bacterial species commonly isolated from brood comb honey (columns 1, 2, 3, 5, 6, 7, 9, 10, 11). Recombinantly expressed SplA served as positive control and culture medium as negative control. (**E**) α-*Pl*CBP49 mAbs and α-recSplA mAbs tested with EFB causing (*M. plutonius*) and associated bacteria (*P. alvei*, *E. faecalis*). Native *Pl*CBP49 (columns 1, 2, 3, 4) and recSplA (columns 5, 6, 7, 8) served as positive controls and culture media used (neg. control 1: DSM1582, neg. control 2: MYPGP) as negative controls. In all dot blot assays the reactivity of the α-mouse IgG/IgM antibodies, which were used in the dot blot as a secondary antibody for detecting the mAbs, was also tested as further negative control. Full length membrane pieces are shown. (All tested bacterial strains are listed in Tables 1 and 2).

The dot blot results showed that all three α-*Pl*CBP49 mAbs recognized all *P. larvae* ERIC I strains (Fig. 1A, columns 1, 4, 7) although signal intensity varied. In contrast, a clear positive signal for all eight *P. larvae* ERIC II strains was only obtained with α-*Pl*CBP49 mAb1 (Fig. 1A, column 2), while the α-*Pl*CBP49 mAb2 (Fig. 1A, column 5) and α-*Pl*CBP49 mAb3 (Fig. 1A, column 8) showed weak signals or no signal at all for four of the eight tested *P. larvae* ERIC II strains (strain 00-1163 in row B, strain 02–0009 in row C, strain 03-0522 in row F, and 03-0525 in row G). The purified antigen *Pl*CBP49 served as positive control (Fig. 1A, row A in columns 3, 6, 9), while culture medium (Fig. 1A, row B in columns 3, 6, 9) served as negative control. None of the three α-*Pl*CBP49 mAbs showed cross-reactivity with any of the tested non-*P. larvae* bacterial species (Table 2) associated with honey bees and commonly isolated from brood comb honey^25^ (Fig. 1B, columns 1, 2, 3, 5, 6, 7, 9, 10, 11). The antigen *Pl*CBP49 as positive control was clearly detected (Fig. 1B, row A in columns 4, 8, 12).

For the tested α-SplA mAbs, the dot blot results showed that all three mAbs gave strong signals for all tested strains of *P. larvae* ERIC II (Fig. 1C, columns 2, 5, 8) although they also weakly recognized some ERIC I strains (Fig. 1C, columns 1, 4, 7). The recombinantly expressed antigen recSplA served as positive control (Fig. 1C, row A in columns 3, 6, 9), while culture medium (Fig. 1C, row B in columns 3, 6, 9) served as negative control. None of the three α-SplA mAbs cross-reacted with any of the tested non-*P. larvae* bacterial species (Table 2) isolated from brood comb honey (Fig. 1D, columns 1, 2, 3, 5, 6, 7, 9, 10, 11), while a strong signal was obtained with the antigen recSplA as positive control (Fig. 1D, row A in columns 4, 8, 12). In all dot blot assays, incubation with only the secondary polyclonal α-mouse antibodies (α-mouse IgG/IgM), which were used as the second antibody in the dot blot to detect the mouse-derived mAbs, served as an additional negative control (Fig. 1A, C, columns 10–12; Fig. 1B, D, columns 13–16).

*M. plutonius*, the causative agent of EFB^26^, as well as the saprophytic bacteria *P. alvei* and *E. faecalis* often associated with EFB^29^ did also not cross-react with the tested antibodies (Fig. 1E). Again, purified *Pl*CBP49 and recombinantly expressed recSplA served as positive controls, the culture media used (DSM1582, MYPGP) as negative controls, and incubation with the second antibody as additional negative control.

Therefore, dot bot analyses proved that α-*Pl*CBP49 mAb1 and α-SplA mAb2 are highly specific for the detection of the species *P. larvae* and the genotype *P. larvae* ERIC II, respectively. This made these two antibodies promising candidates for capture antibodies in the development of immune assays for the detection of and genotype differentiation within the species *P. larvae*. The remaining four antibodies, α-*Pl*CBP49 mAb2 and mAb3 and α-SplA mAb1 and mAb3, have demonstrated sufficient specificity in the dot blot analyses for being potential detector antibodies in the development of *P. larvae* directed immune assays.

**Fig. 2:**
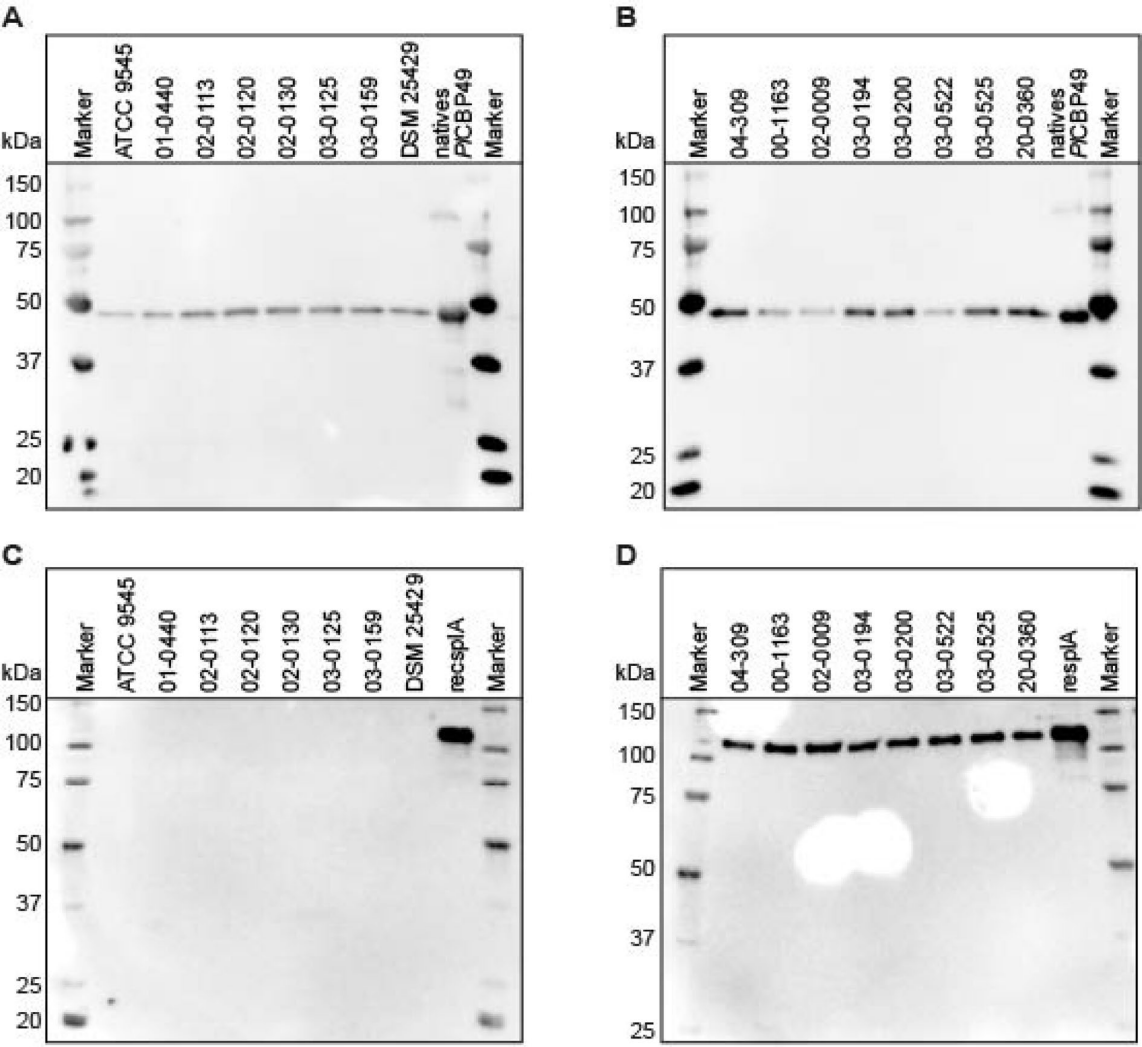
Evaluation of antibody specificity by western blot. Testing of α-*Pl*CBP49 mAb1 with eight different strains of *P. larvae* ERIC I (**A**) and eight different strains of *P. larvae* ERIC II (B); native *Pl*CBP49 served as a positive control. Testing of α-SplA mAb2 with eight different strains of *P. larvae* ERIC I (**C**) and eight different strains of *P. larvae* ERIC II (**D**); recSplA served as a positive control. The Precision Plus Protein WesternC Blotting Standard (Bio-Rad, Feldkirchen, Germany) served as a molecular weight marker for all blots. The original blots in full size are presented in supplementary Figure S2.

To further confirm the specificity of the two candidate capture antibodies, α-*Pl*CBP49 mAb1 and α-SplA mAb2, western blot analysis was performed (Fig. 2) with the same eight strains each of *P. larvae* ERIC I and ERIC II (Table 1) already used in dot blot analysis. The results obtained demonstrated that α-*Pl*CBP49 mAb1 detected a single band migrating at the anticipated molecular weight for *Pl*CBP49 (~ 49 kDa) in all tested strains of *P. larvae* ERIC I (Fig. 2A) and *P. larvae* ERIC II (Fig. 2B). The purified antigen *Pl*CBP49 served as positive control and additional molecular weight marker (Fig. 2A, B). The western blot results obtained with α-SplA mAb2 did not show any signal in the *P. larvae* ERIC I strains tested (Fig. 2C), but demonstrated a single band migrating at the anticipated molecular weight of SplA (~ 114 kDa) in the tested strains of *P. larvae* ERIC II (Fig. 2D). The recombinantly expressed antigen recSplA served as positive control and additional molecular weight marker (Fig. 2C, D). Thus, western blot analysis of the antibodies α-*Pl*CBP49 mAb1 and α-SplA mAb2 confirmed that they exhibit a high specificity for the antigens *Pl*CBP49 or SplA expressed by all tested strains of the species *P. larvae* or only by the genotype *P. larvae* ERIC II, respectively.

### Validation of the sandwich ELISAs for the detection and differentiation of *P. larvae*

Sandwich-ELISAs based on the characterized antibodies were developed according to standard methods by fzmb GmbH. The sandwich ELISA detecting the species *P. larvae* is based on the α-*Pl*CBP49 mAb1/α-*Pl*CBP49 mAb2 pairing with a given (fzmb GmbH) LOD for *Pl*CBP49 of < 0.4 ng/ml as determined by titrating the antigen. The sandwich ELISA developed for genotype differentiation within the species *P. larvae* is based on the antibody pair α-SplA mAb2/α-SplA mAb3 with a given (fzmb GmbH) LOD for SplA of approx. 4.7 ng/ml as determined by titrating the antigen.

To evaluate the specificity and the sensitivity of the sandwich ELISAs provided by the manufacturer (fzmb GmbH) for detecting the species *P. larvae* (*Pl*CBP49-ELISA) or specifically detecting the genotype ERIC II (SplA-ELISA) in infected larvae, we tested both ELISAs with larvae experimentally infected with *P. larvae* ERIC I (*n* = 50) or *P. larvae* ERIC II (*n* = 50) or non-infected larvae (*n* = 50). The *Pl*CBP49-ELISA detected 44 of 50 ERIC I-infected and 45 of 50 ERIC II-infected larvae, hence achieved a sensitivity of 89% (95% CI 81–94) for all *P. larvae*-positive samples (ERIC I- and II-infected larvae). All non-infected larvae gave negative results; therefore, the *Pl*CBP49-ELISA has a specificity of 100% (95% CI 93–100) (Table 3). Using the SplA-ELISA, only three of the tested 50 *P. larvae* ERIC II-infected larvae were not detected, resulting in a sensitivity of 94% (95% CI 84–98) for *P. larvae* ERIC II-infected larvae. The SplA-ELISA did not detect a single larva infected with *P. larvae* ERIC I or a non-infected larva, i.e. there were no false-positive and 100% true-negative results in both cases. Therefore, it has a specificity of 100% (95% CI 96–100) (Table 3). Considering all infected larvae (*n* = 100), the overall accuracy of the SplA-ELISA for the correct identification of *P. larvae* ERIC II and, hence, for the differentiating between the genotypes ERIC I and ERIC II was 97% (95% CI 92–99) (Table 3).

**Table 3:** Specificity, sensitivity and accuracy of the immunoassays (ELISA, LFA) developed for the detection of the species *P. larvae* and the differentiation between the *P. larvae* genotypes ERIC I and ERIC II.

	Specificity (%)	Sensitivity (%)	Accuracy of genotype differentiation (%; within positive samples)
*Pl*CBP49-ELISA (exp. inf. larvae)	100	89	Not applicable
SplA-ELISA (exp. inf. larvae)	100	94	97%
Duplex-LFA (exp. inf. larvae)	96	95	91%
Duplex-LFA (field samples)	100	99	91%

**Fig. 3:**
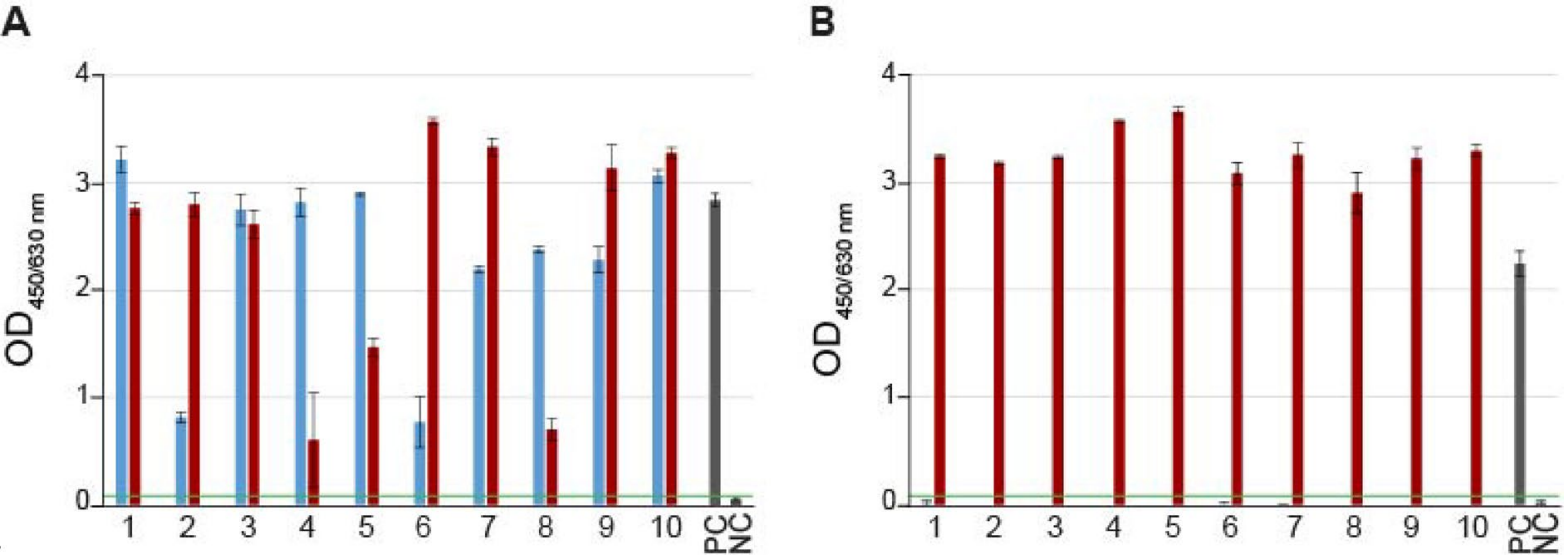
ELISA assay results. (**A**) Representative results of the *Pl*CBP49-ELISA assay for the detection of the species *P. larvae* in *P. larvae* ERIC I-infected (blue) and *P. larvae* ERIC II-infected larvae (red). (**B**) Representative results of the SplA-ELISA assay for the differentiation between *P. larvae* ERIC I-infected (blue) and *P. larvae* ERIC II-infected larvae (red). The bar charts show the OD_450/630 nm_ values (mean ± SD) of ten larval samples per genotype with three measured wells per sample. The green line shows the cut-off value (0.1) for a positive detection. The assays were validated using the ELISA kit positive (PC) and negative (NC) controls. OD Signals for all non-infected larvae in both assays were < 0.1, and are therefore not shown here.

The *Pl*CBP49-ELISA (Fig. 3A) and the SplA-ELISA (Fig. 3B) also generated sufficiently high OD signals to obtain clear results. The findings indicate that both ELISA assays are reliably detecting their target (species *P. larvae* or genotype ERIC II), resulting in a 100% specificity in detecting *P. larvae* and a 97% accuracy in differentiating between the genotypes.

### Duplex-LFA for detection and differentiation of *P. larvae*

**Fig. 4:**
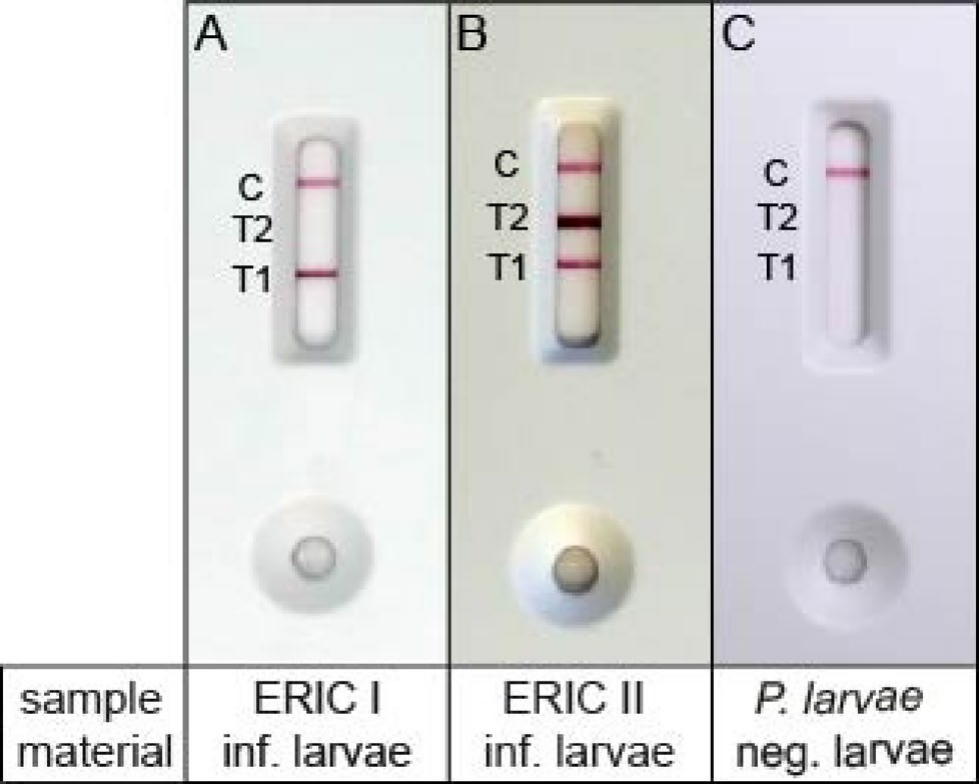
Visual interpretation of the duplex-LFA. (**A**) A positive result for *Pl*CBP49 (test line T1 and control line C appear); (**B**) a positive result for *Pl*CBP49 and SplA (test line T1, test line T2 and control line C appear); (C) a negative result (only the control line C appears). Note, the appearance of test line T1 without test line T2 can be judged as positive for *P. larvae* ERIC I, while the appearance of both test lines T1 and T2, can be judged as positive for *P. larvae* ERIC II.

While the ELISA format is well suited for laboratory analysis of samples, point of care testing (POCT), e.g. testing conspicuous larvae directly at a presumably AFB-diseased colony, needs a different test format like an LFA. Since the ELISA format had shown 100% specificity for both antigens and 89% and 94% sensitivity for the detection of *P. larvae* and *P. larvae* ERIC II (Table 3), respectively, the same antibody pairs were used by the manufacturer (fzmb GmbH) for the construction of a duplex-LFA with a given (fzmb GmbH) LOD for *Pl*CBP49 detection of ≤ 15.63 ng/ml and for SplA detection of < 31.25 ng/ml. The LFA displays three bands: the control line (C) for proving functionality of the LFA, the test line 1 (T1) for detecting *Pl*CBP49 and the test line 2 (T2) for detecting SplA (Fig. 4). Anti-mouse antibodies were immobilized on the membrane at the control line C, while the antibodies α-*Pl*CBP49 mAb1 and α-SplA mAb2 were immobilized as capture antibodies at locations T1 and T2, respectively. The conjugate pad contained the colloidal gold-conjugated antibodies α-*Pl*CBP49 mAb2 and α-SplA mAb3 as detectors. A visible signal at the control line C validates the assay. A visible signal at T1 is indicative for the presence of *Pl*CBP49 in the sample and is a positive result for the species *P. larvae*. If T1 is positive while T2 remains negative, indicating the absence of the *P. larvae* ERIC II- specific S-layer protein SplA, the result corresponds to the specific detection of *P. larvae* ERIC I (Fig. 4A). An additional signal visible at T2 indicates the presence of both *Pl*CBP49 and SplA in the sample and counts as a positive result for *P. larvae* ERIC II (Fig. 4B). In the event of the application of a *P. larvae* negative sample, the sole visible line should be the control line C (Fig. 4C).

Specificity and sensitivity of this duplex-LFA was initially evaluated with 197 larvae experimentally infected with *P. larvae* (*n* = 96 for *P. larvae* ERIC I; *n* = 101 for *P. larvae* ERIC II), and non-infected larvae (*n* = 101), the latter obtained from the negative control group of the experimental infection assays. The duplex-LFA detected the species *P. larvae* in 187 of the tested 197 larvae infected with *P. larvae* (infected with *P. larvae* ERIC I and ERIC II), which corresponds to a sensitivity of 95% (95% CI: 91–97). Among the 101 non-infected control larvae, four showed false-positive results (one or both test lines visible), resulting in a specificity of 96% (95% CI 90–98). Regarding the accuracy of genotype differentiation, 83/96 of the *P. larvae* ERIC I infected larvae and 96/101 of the *P. larvae* ERIC II infected larvae were correctly identified. This resulted in an overall accuracy of the duplex-LFA for correctly differentiating between the two genotypes in experimentally infected larvae of 91% (95% CI: 86–94) (Table 3).

**Fig. 5:**
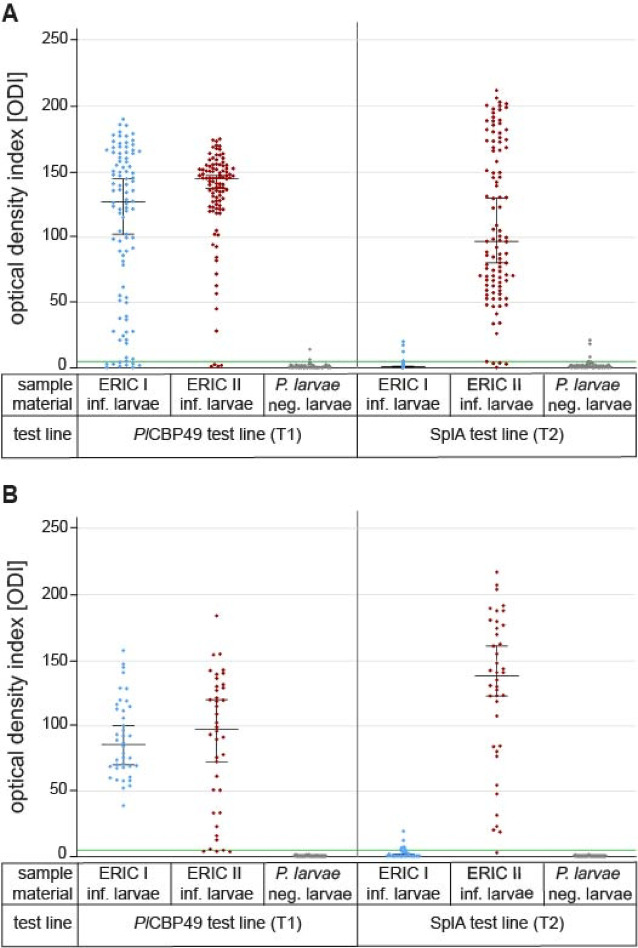
Colorimetric assessment of the signal strength. (**A**) Determination of the signal strength with the Cube Reader (ODI) of experimental infected larvae in the *P. larvae* duplex-LFA. Results shown for *P. larvae* ERIC I-infected (blue), *P. larvae* ERIC II-infected (red) and non-infected, *P. larvae* negative larvae (grey). The scatter plots show the data of 96–101 samples per sample material as well as the median (95% CI of median) of the samples per group. (**B**) Determination of the signal strength with the Cube Reader (ODI) of field samples from AFB-outbreaks in the *P. larvae* duplex-LFA. Results shown for *P. larvae* ERIC I-infected (blue), *P. larvae* ERIC II-infected (red) and non-infected, *P. larvae* negative larvae (grey). The scatter plots show the data of 40 samples per sample material as well as the median (95% CI of median) of the samples per group. The green line shows the cut-off value (ODI = 5) for positive detection.

In order to confirm the visual reading and enhance measurement objectivity, all test results were quantified colorimetrically using the Cube-Reader^34^ (opTricon GmbH) with the signal intensity given in optical density indices (ODI). The median of the signal strength for the *Pl*CBP49 test line (T1) was 126.7 ODI (95% CI: 101.9–144.2) for ERIC I-infected larvae, 144.2 ODI (95% CI 136.8–146.7) for ERIC II-infected larvae, and 0.0 ODI (95% CI 0.0–0.3) for non-infected larvae (Fig. 5A). The median of the signal strength for the SplA test line (T2) was 0.7 ODI (95% CI 0.4–1.1) for ERIC I-infected larvae, 96.2 ODI (95% CI 79.6–128.9) for ERIC II-infected larvae, and 0.8 ODI (95% CI 0.4–1.1) for non-infected larvae (Fig. 5A).

Since the performance of the duplex LFA was convincing in the diagnosis of experimentally infected larvae (Table 3), the test was finally evaluated using the truly relevant samples, namely field samples of larvae infected with *P. larvae* ERIC I and ERIC II (*n* = 40 each), which were taken from brood combs from two different, officially diagnosed AFB-outbreaks. *P. larvae*-negative, non-infected larvae (*n* = 40) were used as negative controls. The duplex-LFA detected *P. larvae* in 79/80 *P. larvae*-infected larvae (*P. larvae* ERIC I- and ERIC II-infected larvae) corresponding to a sensitivity of 99% (95% CI 93–100). The observed differences in test sensitivity between experimentally infected larvae (95%) and field samples (99%) were not statistically significant (chi-square test, *p* > 0.05). None of the non-infected larvae gave a signal at the test lines T1 or T2, hence all were correctly diagnosed as non-infected, corresponding to a specificity of 100% (95% CI 91–100). Regarding the accuracy of genotype differentiation, 34/40 of the *P. larvae* ERIC I infected larvae and 39/40 of the *P. larvae* ERIC II infected larvae were correctly identified. This resulted in an overall accuracy of the duplex-LFA for correctly differentiating between the two genotypes in field samples of 91% (95% CI 83–96) (Table 3).

The visual reading was again confirmed and measurement objectivity enhanced by colorimetric assessment of the signal strength at the test lines T1 and T2 using the Cube-Reader. The median signal height of all infected samples for the *Pl*CBP49 test line (T1) was 85.0 ODI (95% CI 69.6–99.6) for ERIC I-infected larvae, 96.95 ODI (95% CI 71.9-119.2) for ERIC II-infected larvae, and 0.0 ODI (95% CI 0.0–0.0) for non-infected larvae (Fig. 5B), while the median signal height for the SplA test line (T2) was 0.0 ODI (95% Cl: 0.0-1.5) for ERIC I-infected larvae, 137.3 ODI (95% CI 122.1-160.4) for ERIC II-infected larvae, and 0.0 ODI (95% CI 0.0–0.0) for non-infected larvae (Fig. 5B).

## Discussion

We used our knowledge on the virulence factors of *P. larvae* for the development of two immunoassays, an ELISA and an LFA, as novel diagnostic tools for the detection of *P. larvae*, the causative agent of AFB^5^. Both assays are based on the immunodetection of the virulence factors *Pl*CBP49^17^ and SplA^19^. The reason for selecting these two antigens was that, despite all the potential variability of *P. larvae*, these two proteins are so important for pathogenicity and virulence that it can be assumed with certainty that *Pl*CBP49 is present in all virulent AFB-causing *P. larvae* strains and that SplA is additionally present in *P. larvae* ERIC II strains. While the ELISA is designed for laboratory use only, the LFA is primarily intended as a point-of-care test to aid the clinical diagnosis of AFB.

The laboratory diagnosis of *P. larvae* has been based for decades on classical microbiological techniques like cultivation of *P. larvae* from sample material on different culture plates (e.g. Columbia sheep blood agar plates^35^) followed by further microbiological (e.g. induction of sporulation to initiate giant whip formation^36^), biochemical analyses (e.g. catalase test^22^), and molecular techniques (e.g. DNA fingerprinting^37^) for the definite identification of *P. larvae*. PCR-based diagnostic methods became possible with the reclassification of the species *P. larvae*, which showed that the species does not comprise two differentially pathogenic subspecies that need to be distinguished^5^. It was therefore not necessary to have PCR-tests available that can differentiate between *P. larvae* subspecies^38^; instead, it was sufficient to detect the species. However, the reclassification of the species *P. larvae* revealed that determining the ERIC-genotype of the *P. larvae* strain identified in sample material is advisable since the differences in virulence need to be considered when deciding on appropriate mitigation strategies. The ERIC genotype is still identified using the original repPCR^5,6^, although multi-locus sequence typing (MLST) and MALDi-ToF analysis can also provide information about the genotype^7,39^.

Immunoassays are among the most commonly used tools for pathogen detection and also suitable for point-of-care testing (for a recent review see^40^). The core principle of immunoassays is the use of antibodies that bind specifically to a target protein. Ideally, a pair of antibodies is available, with one antibody serving as a capture antibody and the other as a detection antibody for the captured antigen (for recent reviews see^41,42^). Surprisingly, immunoassays have not yet become widely accepted in *P. larvae* diagnostics, despite the fact that the first attempts to develop antibody-based techniques for detecting *P. larvae* date back to the 1970s^43,44^. The first enzyme-linked immunosorbent assay (ELISA), an indirect ELISA, was developed using monoclonal antibodies already 35 years ago^45^, but it never became a standard method in *P. larvae* diagnostics. A lateral flow assay (LFA) for *P. larvae* is commercially available since about 20 years. However, to the best of our knowledge, no peer-reviewed publications on the reliability, sensitivity, and specificity of this LFA are available. Moreover, when the LFA was evaluated in a recent doctoral thesis, it was shown that it was not capable of detecting *P. larvae* ERIC II^46^.

To close this gap in the immunoassay-based diagnosis of *P. larvae*, we pursued a relatively simple, straightforward approach: Based on our previous work on the virulence factors of *P. larvae*^10,15,17–20,47−50^ we selected *Pl*CBP49^17^ and SplA^19^ as suitable antigens and had monoclonal antibodies against these antigens commercially produced. Since *Pl*CBP49 is the key virulence factor of the *P. larvae* species^17^, we hypothesized that antibodies against this protein will specifically recognize all virulent members of the *P. larvae* species. SplA is exclusively expressed by *P. larvae* ERIC II^19,20^, therefore, we hypothesized that antibodies against this protein will not recognize members of the *P. larvae* ERIC I. When testing the specificity of the antibodies obtained, our hypotheses were confirmed: α-*Pl*CBP49 antibodies detected all *P. larvae* strains, while α-SplA antibodies specifically detected *P. larvae* ERIC II strains. We next selected suitable antibody pairs for the commercial development and production of immunoassays (fzmb GmbH), which we then evaluated with AFB-diseased larvae.

The ELISA test system developed for laboratory use, when tested with experimentally infected larvae, did not falsely diagnose non-infected larvae as infected, resulting in a specificity of 100% (95% CI 93–100), and detected *P. larvae* or *P. larvae* ERIC II with a sensitivity of 89% (95% CI 81–94) or 94% (95% CI 84–98), respectively. These results are a promising start for further large-scale field validations, which are now set to follow. Tests will be carried out using real-world samples from various AFB outbreaks in order to verify the test’s sensitivity, specificity, and accuracy, and to identify potential issues or limitations of the ELISA that have not been apparent so far.

Since, to our knowledge, there are currently no ELISA tests available for the diagnosis of *P. larvae*, let alone for the differentiation between the ERIC I and ERIC II genotypes, we cannot discuss the advantages and disadvantages of the newly developed ELISA compared to similar tests. We can only conclude that this ELISA test system will be a valuable diagnostic tool once it has been approved, especially if a laboratory focuses on relatively inexpensive methods that deliver rapid results and can be easily automated, thereby reducing manual work and increasing throughput.

A great advantage of the developed ELISA test system is that it not only detects the species *P. larvae* via the *Pl*CBP49-ELISA but also enables the easy differentiation between the two genotypes via the SplA-ELISA: Samples that are positive for *Pl*CBP49 but not for SplA are diagnosed as *P. larvae* ERIC I, whereas samples that additionally react positively for SplA are identified as *P. larvae* ERIC II. This easy-to-obtain diagnostic result on the *P. larvae* genotype causing an outbreak can be used to inform the mitigation measures in order to decide on the most appropriate control method adapted to the genotype.

Our primary goal was to develop a duplex-LFA as a point-of-care test for the direct, one-step diagnosis of *P. larvae* and the differentiation between the two genotypes ERIC I and ERIC II in a suspected honey bee colony. This would support the clinical diagnosis of AFB, which has so far been based on finding clinical symptoms during a visual inspection of the brood combs, i.e., finding the remains of larvae that have died from AFB and remained as viscous masses and scales in the brood cells^51^. However, such symptoms do not occur exclusively in AFB-diseased colonies, but can also occur in a similar manner whenever saprophytic bacteria decompose dead larvae or pupae, for instance in the course of other brood diseases such as European foulbrood^29^ or ABPV infections of pupae^52^. Therefore, any visual differential diagnosis must be confirmed by identification of *P. larvae* in suitable test material. To this end, suspicious brood combs, diseased larvae, or ropy masses are sent to a laboratory, where the pathogen is detected and identified, for example, by species-specific PCR^53,54^ or MALDI-ToF^39^, often performed after culturing the pathogen. This procedure is time-consuming. Therefore, a point-of-care test that can identify and differentiate *P. larvae* directly at the colony would significantly improve and accelerate the diagnosis of AFB.

While the *P. larvae* duplex LFA presented here did not achieve 100% specificity in tests with experimentally infected larvae, it did so in tests with real samples, i.e., with diseased or dead larvae that had died from infection with *P. larvae*, or with uninfected larvae (both determined by PCR) from officially confirmed AFB-diseased colonies. Both test lines, T1 for the detection of *Pl*CBP49 (indicative for *P. larvae* ERIC I and ERIC II) and T2 for the additional detection of SplA (indicative only for *P. larvae* ERIC II) did not produce any false positive results in non-infected individuals. Hence, specificity of the duplex-LFA for detecting *P. larvae* infection was 100% (95% CI 91–100), which is important considering the implications of positive diagnoses (ban of the apiary, setting up a restricted area, analysing all colonies in this area).

Sensitivity of the LFA when tested with dead or degraded larvae from diseased colonies was 99% (95% CI 93–100). In order to classify these values obtained for sensitivity and specificity in detecting *P. larvae*, we selected a recently published systematic literature review from the many studies on LFA performance in detecting infectious agents for comparison. This review evaluated 24 articles that had assessed the sensitivity and specificity of SARS-CoV-2 LFAs from a total of eight manufacturers^55^. The sensitivity of these SARS-CoV-2 LFAs ranged from 37.7% (95% CI 30.6–45.5) to 99.2% (95% CI 95.5–99.9). Specificity of these LFAs was always over 92%, with eleven studies even having a specificity of 100%. From these values we conclude that the performance of the newly developed duplex-LFA for the detection of *P. larvae*, with a specificity of 100% and a sensitivity of 99% in tests with sample material from AFB-diseased colonies, can compete with the performance of other LFAs that detect infectious agents. We even expect that in further large-scale field validations, which must now follow and will include more AFB outbreaks and diseased colonies, achieving 100% sensitivity should be possible by testing more than one larva per suspect colony.

The newly developed ELISA and duplex-LFA presented here do not only detect both *P. larvae* genotypes, ERIC I and ERIC II, but can also distinguish between the two genotypes. Hence, these novel immunoassays represent a considerable step forward in the diagnosis of AFB. Especially a *P. larvae*-LFA not producing any false positive results, achieving as close to 100% sensitivity as possible, and differentiating between the genotypes with an accuracy of 91% is expected to become a valuable and highly accepted diagnostic tool in the field. It will improve the clinical diagnosis and combine the clinical diagnosis with pathogen detection at the point of care thus speeding up the entire diagnostic process and allowing measures to be decided upon and implemented at an earlier stage. Since speed is a crucial factor in combating epizootics, improved and faster diagnosis, leading to early intervention, will improve the control of AFB.

## Supplementary Information

Below is the link to the electronic supplementary material.

Supplementary Material 1

## Data Availability

The authors declare that the data supporting the findings of this study are available within the paper. The presented ELISA and LFA tests are commercially available through fzmb GmbH, Germany. Should any raw data files be needed in another format, these are available from the corresponding author upon reasonable request.
