# Supplementary material for: Immunoassays for the detection and differentiation of Paenibacillus larvae, the etiological agent of American foulbrood (AFB) in honey bees
Source: Sci Rep. 2026 Jan 20;16:2635. doi: 10.1038/s41598-026-35590-7 (PMC12823653; doi:10.1038/s41598-026-35590-7)
Supplement: Supplementary file 1 — Supplementary Material 1 [file 41598_2026_35590_MOESM1_ESM.pdf]

Supplementary material for:

Immunoassays for the detection and differentiation of *Paenibacillus larvae*, the etiological agent of American foulbrood (AFB) in honey bees

Antonia Reinecke[1], Josefine Göbel[1], and Elke Genersch[1,2]

[1] Institute for Bee Research, Department of Molecular Microbiology and Bee Diseases, 16540 Hohen Neuendorf, Germany
[2] Freie Universität Berlin, Department of Veterinary Medicine, Institute of Microbiology and Epizootics, 14163 Berlin, Germany

* Corresponding author: Elke Genersch, Institute for Bee Research, Friedrich-Engels-Str. 32, 16540 Hohen Neuendorf, Germany,
telephone: ++49 (0)3303 293833
fax: ++49 (0)3303 293840
E-mail: elke.genersch@hu-berlin.de, elke.genersch@fu-berlin.de

**primer name**	**primer sequences**	**Used for**	**Reference**
NdeI_SplA	5'-GTG AAT CAT ATG ATG AGG AAT ATG GGC TCC G-3'	recombinant production	this study
XhoI_SplA	5'-TTA GTT CTC GAG TTA AAG GTT TTT AAC AAG ATT ACC AGC-3'		
S-layer-F2	5'-ACT ATC AGC AAA TCG TTA TTG AAG G -3'	sequence analysis	Poppinga et al. 2012
S-layer-F3	5'-TAT TAA ACC TGG AAA AGT AGA TGT CC -3'		Poppinga et al. 2012
S-layer-F5	5'-AAG ATT TAA TTG AAA CTC TTA ATG CAG-3'		Poppinga et al. 2012
S-layer-R1	5'-CTG TTT TTT CGT TAA GCA TGG TT -3'		Poppinga et al. 2012
S-layer-R2	5'-TCA ACT GTT GTT GCA CCG G -3'		Poppinga et al. 2012
S-layer-R4	5'- AAT CCG CAG AAC CTT TAG CA -3'		Poppinga et al. 2012
T7	5'-TAA TAC GAC TCA CTA TAG GG-3'		Eurofins Genomics
T7_term	5'-CTA GTT ATT GCT CAG CGG T-3'		Eurofins Genomics

Supplementary Table S1: Primers used for recombinant production of SplA and sequence analysis of pET28(+)_*spl*A.

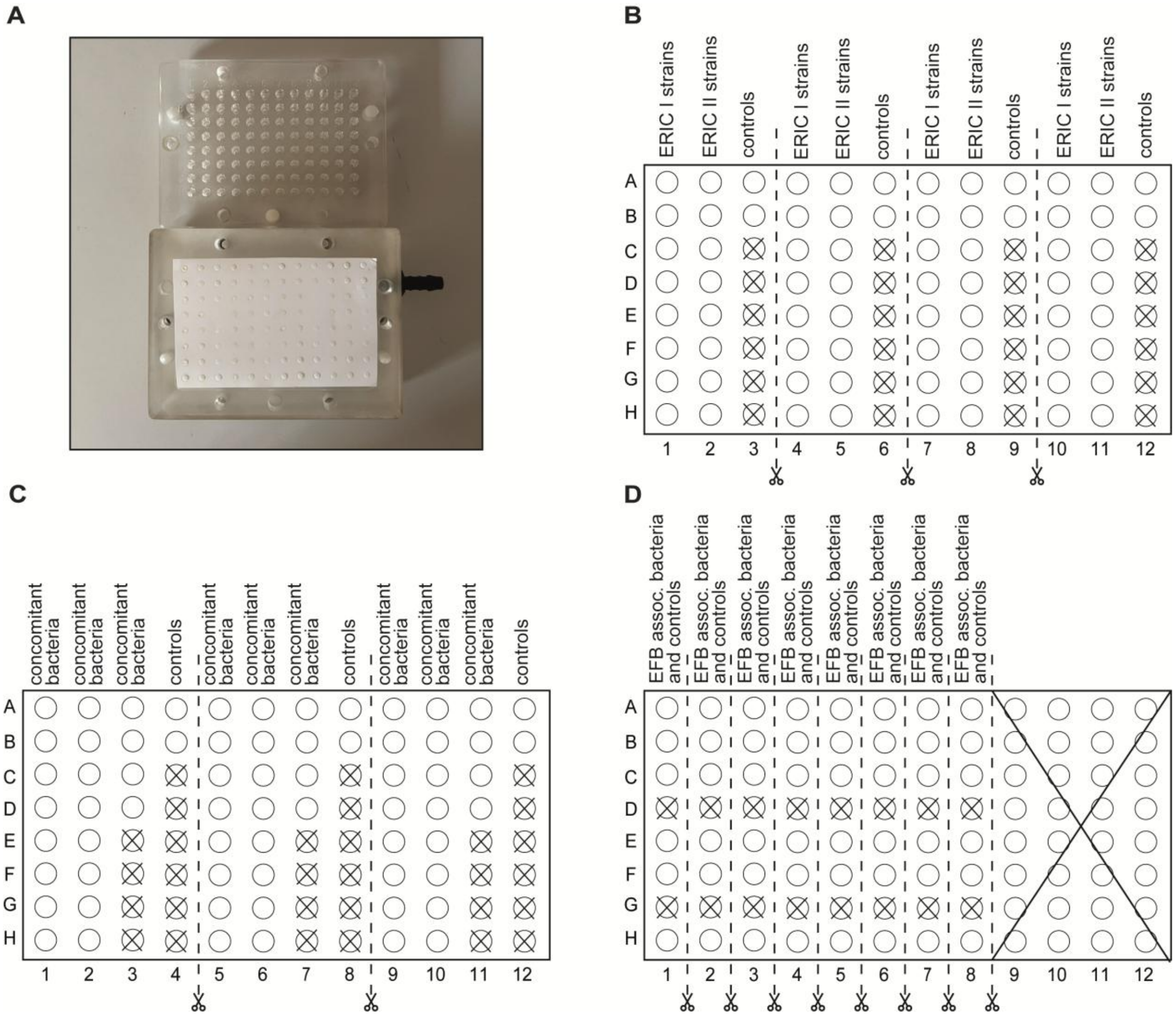

Figure S1: **Loading scheme dot blot.** (A) Picture of 96-pocket transfer device with loaded membrane. (B) Loading scheme of dot blot for testing *P. larvae* ERIC I (n=8) and ERIC II (n=8) strains. (C) Loading scheme of dot blot for testing concomitant bacteria (n=20). (D) Loading scheme of dot blot for testing EFB causing and associated bacteria (n=3). The Membranes were cut vertically along the dotted lines, after samples were blotted onto the membrane. Spots marked with an X were not loaded with sample material.

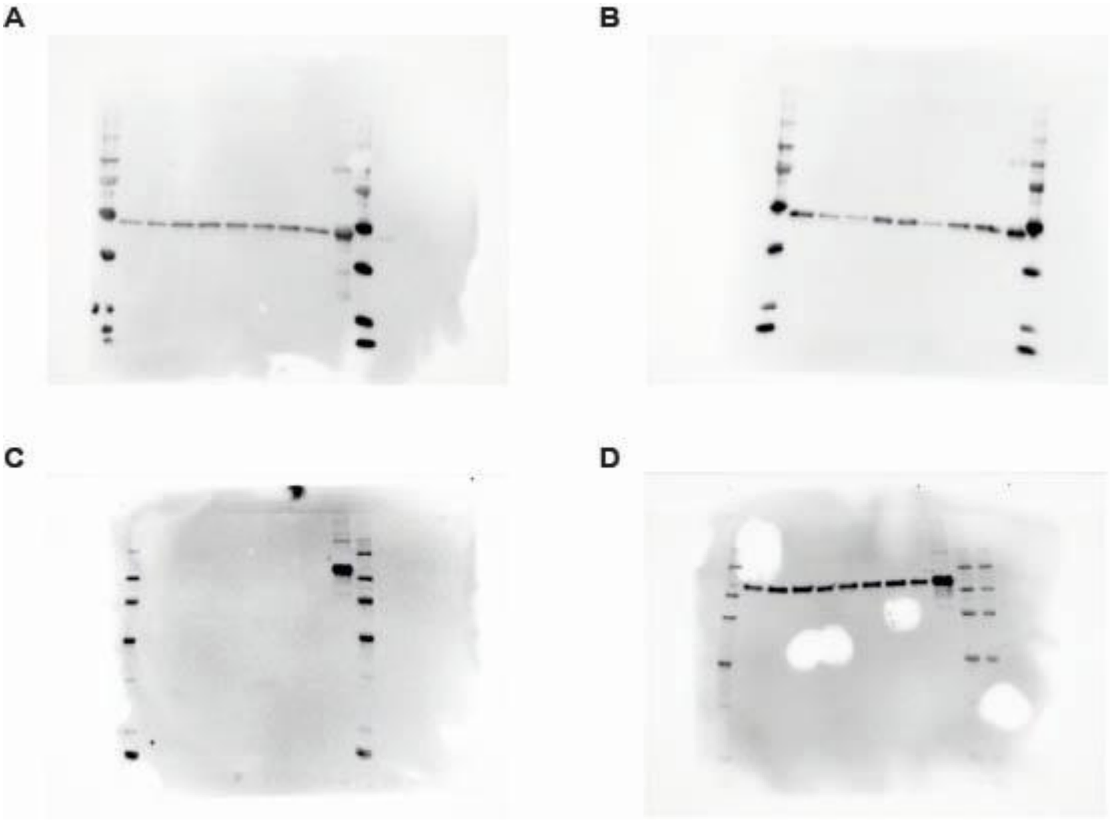

Figure S2: **Original full length western blots shown in Figure 1.** (Evaluation of antibody specificity by western blot. Testing of α-*Pl*CBP49 mAb1 with eight different strains of *P. larvae* ERIC I (A) and eight different strains of *P. larvae* ERIC II (B); native *Pl*CBP49 served as a positive control. Testing of α-SplA mAb2 with eight different strains of *P. larvae* ERIC I (C) and eight different strains of *P. larvae* ERIC II (D); recSplA served as a positive control. The Precision Plus Protein WesternC Blotting Standard (Bio-Rad, Feldkirchen, Germany) served as a molecular weight marker for all blots.)
